# Crosstalk between Activated Microglia and Neurons in the Spinal Dorsal Horn Contributes to Stress-induced Hyperalgesia

**DOI:** 10.1038/srep39442

**Published:** 2016-12-20

**Authors:** Jian Qi, Chen Chen, Qing-Xi Meng, Yan Wu, Haitao Wu, Ting-Bao Zhao

**Affiliations:** 1 Department of Spinal Cord Injury and Rehabilitation, The General Hospital of Jinan Military Command, Jinan, 250031, China; 2 Department of Pharmacy, The Second Hospital of Shandong University, Jinan, 250031, China; 3 Department of Neurobiology, Institute of Basic Medical Sciences, Beijing, 100850, China; 4 Co-innovation Center of Neuroregeneration, Nantong University, Nantong, Jiangsu, 226001, China

## Abstract

Stress has been shown to enhance pain sensitivity resulting in stress-induced hyperalgesia. However, the underlying mechanisms have yet to be elucidated. Using single-prolonged stress combined with Complete Freund’s Adjuvant injection model, we explored the reciprocal regulatory relationship between neurons and microglia, which is critical for the maintenance of posttraumatic stress disorder (PTSD)-induced hyperalgesia. In our assay, significant mechanical allodynia was observed. Additionally, activated neurons in spinal dorsal horn were observed by analysis of Fos expression. And, microglia were also significantly activated with the presence of increased Iba-1 expression. Intrathecal administration of c*-fos* antisense oligodeoxynucleotides (ASO) or minocycline (a specific microglia inhibitor) attenuated mechanical allodynia. Moreover, intrathecal administration of c-*fos* ASO significantly suppressed the activation of neurons and microglia. Interestingly, inhibition of microglia activation by minocycline significantly suppressed the activation of both neurons and microglia in spinal dorsal horn. P38 inhibitor SB203580 suppressed IL-6 production, and inhibition of IL-6 receptor (IL-6R) activation by tocilizumab suppressed Fos expression. Together, our data suggest that the presence of a “crosstalk” between activated microglia and neurons in the spinal dorsal horn, which might contribute to the stress-induced hyperactivated state, leading to an increased pain sensitivity.

Chronic pain occurs after damage or dysfunction of peripheral and central sensory pathways (neuropathic pain), or after tissue inflammation (inflammatory pain)^1^. Chronic inflammatory pain is associated with the subcutaneous (s.c.) injection of complete Freund’s adjuvant (CFA) into a hindpaw^2^,^3^. Clinical observations suggest stressful stimuli promote an increase in pain sensitivity, leading to the exacerbation of existing pain^4^,^5^. These phenomena are collectively termed stress-induced hyperalgesia (SIH)^6^,^7^. Understanding how stress affects the development and severity of pain provides a potential application for therapies in a variety of pain syndromes.

Glial cells, including microglia and astrocytes, play important roles in immunosurveillance, monitoring cellular debris, apoptotic cells, alterations in neuronal phenotypes and synaptic homeostasis^8^. Emerging research in chronic pain animal models has shown that microglia play a vital role in the initiation or maintenance of hyperalgesia and allodynia^9^. Various physiological or noxious stimuli have been shown to increase the expression of ionized calcium-binding adapter molecule-1 (Iba-1), which is used as a marker for microglia^10^. Several reports have demonstrated that microglia are responsive to environmental stressors in the spinal cord^2^,^11^. In addition, microglia are found to exist in a hyperactivated state following stress. This effect is likely to lead to the potentiation of immune responses, thereby promoting peripheral stimulation^12^.

Depending on the nature and duration of the stressor, stress hormones may either inhibit the production of pro-inflammatory cytokines or boost immune responses via peripheral production of pro-inflammatory cytokines, including IL-1β, or IL-6^13^. Previous studies showed that activated microglia could contribute to the enhanced pain-like state experienced by stressed mice. It is possible that microglia could release pro-inflammatory cytokines and brain derived neurotrophic factor (BDNF), leading to enhance neuronal excitability^14^. Reactive microglia show increased phosphorylation of mitogen-activated protein (MAP) kinases-p38 MAPK and extracellular signal-regulated kinase (ERK1/2)-which are in part responsible for the secretion of cytokines by microglia and for their proliferation^15^.

Post-traumatic stress disorder (PTSD) is an anxiety and stress disorder with severe psychological consequences following exposure to stressful events for associated individuals^16^. It has been reported that PTSD can result in an increase in pain sensitivity^17^. However, the underlying mechanism that underpins this sensitivity is not fully understood. It has been suggested that chronic pain develops as a consequence of enhanced neuro-immune signaling and central sensitization in the spinal cord^18^. Hence, we hypothesize that activated microglia and neurons are involved in a “crosstalk” regulatory interaction that contributes to PTSD-induced hyperalgesia. However, the molecular mechanisms that underpin this interaction remain to be elucidated.

To test this hypothesis, we attempted to negate the effects of activated microglia or neurons to elucidate their individual contributions to stress-induced hyperalgesia. In the current study, single prolonged stress (SPS) was employed to further analyze these effects on PTSD^19^,^20^,^21^. Complete Freund’s adjuvant (CFA) injection was used to promote chronic inflammatory pain^22^. Minocycline was used to inactivate microglia and c*-fos* antisense oligodeoxynucleotides (ASO) was used to disable activated neurons^23^,^24^. We hypothesized that SPS could exacerbate the CFA-induced hypersensitive state. Consequently, we incorporated the use of a model that combined SPS and CFA (referred to as SPS + CFA model). We subsequently monitored the activation of neurons and microglia in SPS, CFA, and SPS + CFA models by determining Fos (a marker for activated neurons) and Iba-1 (a specific protein marker of microglia) expression levels, respectively. The study was to determine the effect of intrathecal (i.t.) injection of c-*fos* ASO or Minocycline on pain behaviors as well as neuron or microglia activation through morphological examination. Moreover, to further determine which downstream cytokines might be involved in crosstalk between microglia and neurons.

## Material and Methods

### Animals

Male Sprague-Dawley rats (weighing 250–300 g) were used in all experiments. All experiments were approved by the Animal Care Committee at the General Hospital of Jinan Military (Jinan, P. R. China) and conformed to the ethical guidelines of the China Council on Animal Care and the guidelines of the International Association for the Study of Pain Committee for Research and Ethical Issues. Animals were housed in plastic cages, and given access to food and water ad libitum under conditions of 22–25 °C ambient temperature using a 12/12 h dark-light cycle (light on at 6:00 and off at 18:00).

### Models

Rats were injected with 50% CFA (50 μl, Sigma, St Louis, MO, USA; 1 mg M. tuberculosis in 0.85 ml mineral oil and 0.15 ml mannide mono-oleate) to generate a chronic inflammatory pain model. SPS was used to generate the PTSD model. This included 2 h restraint stress followed immediately by forced swim test (usually 20 min). The associated rats were allowed to recover for a short period (15 min) and were then exposed to diethylether until unconsciousness was achieved. We used elevated plus maze (EPM) test and the Morris water maze (MWM) to determine the success of the model. Based on these two assays, the success rate of our PTSD model is very high, which is close to 90% (See Supplemental Figs 2 and 3).

### Administration of drugs and surgical procedures

**Figure 1:**
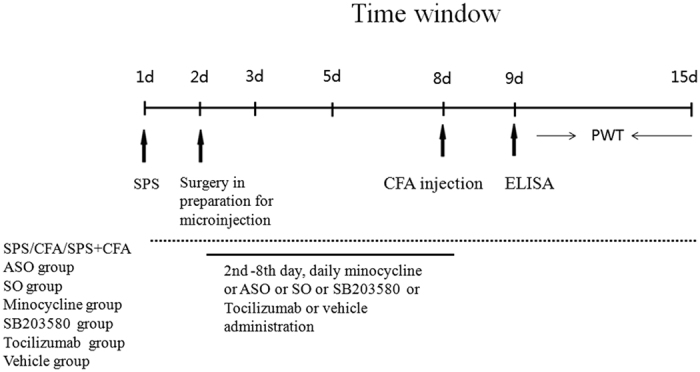
Schematic diagram of experimental design. The diagram depicts the behavioral tests conducted for each group, the time course associated with SPS treatment, the time course for CFA injections, and the duration of time associated with minocycline and ASO administration. SPS, single-prolonged stress; CFA, Complete Freund’sAdjuvant; PWT, paw withdrawal threshold; ELISA, Enzyme-Linked Immunosorbent Assay.

Briefly, rats were exposed to SPS on day 1, and/or underwent CFA administration on day 8 to generate the SPS group, the CFA group and the SPS + CFA group (Fig. 1). A detailed procedure can be found in our previously published study^7^. For c-*fos* ASO administration, the rats were randomly assigned to one of three groups: (1) ASO group: rats underwent c-*fos* ASO (5′-GAA CAT CATGGTCGT-3′) intrathecal administration (dissolved in 5% DMSO, 5 μg/10 μl); (2) SO group: rats underwent c-*fos* sense oligodeoxynucleotide (SO, 5′-ACG ACC ATG ATG TTC-3′) intrathecal administration (dissolved in 5% DMSO, 5 μg/10 μl); (3) DMSO group: rats underwent DMSO (5% DMSO, 10 μl) intrathecal administration. As a potent inhibitor of microglia activation, minocycline (at a concentration of 5 μg/10 μl, dissolved in 5% DMSO, Sigma, St. Louis, MO, USA) was administered by intrathecal administration, using 5% DMSO (10 μl) as a vehicle control. As a potent inhibitor of p38 activation, SB203580 (at a concentration of 5 μg/10 μl, dissolved in 5% DMSO, Sigma, St. Louis, MO, USA) was administered by intrathecal administration, using 5% DMSO (10 μl) as a vehicle control. As a potent inhibitor of IL-6 receptor (IL-6R) activation, tocilizumab (at a concentration of 5 μg/10 μl, dissolved in 5% DMSO, Roche Pharma, S20130020) was administered by intrathecal administration, using 5% DMSO (10 μl) as a vehicle control. The drugs or DMSO were administrated on a daily basis between the second and eighth days (inclusive) prior to CFA administration (performed on day 8). Each group included 18 rats: six rats were studied using behavioral tests, four rats were utilized for immunohistochemical staining, four rats were required to conduct Western blot analysis, and four rats were required to conduct Enzyme-Linked Immunosorbent Assay (ELISA). For intrathecal drug administration, a midline incision (2 cm) was made at the back of the rat at the level of thoracic vertebrae 3–4. A polyethylene tube was directly inserted into the subarachnoid space of the lumbar enlargement. The tube was subsequently released through the rat’s neck skin and positioned subcutaneously in a small pocket between the scapulas. Rats were allowed to recover for a period of 3–5 d before further use. Only the rats judged as neurologically normal were used for follow-on experimentation.

### Behavioral tests

To observe how different treatments affect allodynia, mechanical allodynia was performed double-blindly. These tests were initiated on days 1–15. Mechanical allodynia was examined once daily for each rat. All rats were habituated in the testing room for 15 min prior to behavioral testing. In order to determine evoked reflex responses to mechanical stimuli, rats were placed on an elevated mesh floor covered with inverted plastic boxes (30 × 30 × 50 cm). The threshold for withdrawal of the right hindpaw was tested using Von Frey filaments (Stoelting, Kiel, WI, USA). One of a series of Von Frey filaments with gradually increasing stiffness (2, 4, 6, 8, 10, 15, and 26 g) was applied to touch the plantar surface for 5–6 s. Each filament was used ten times and the paw withdrawal threshold (PWT) was considered the minimal value causing at least six responses. Acute withdrawal, biting, licking, or shaking of the ipsilateral hind limb and vocalization were considered to be positive signs of withdrawal.

### Immunohistochemical staining

Animals were sacrificed on day nine to facilitate immunohistochemical staining of Iba-1 and Fos in spinal cords. The rats were anesthetized by intraperitoneal injection of sodium pentobarbital (50 mg/kg) dissolved in 0.9% (w/v) saline and were perfused transcardially with 150 ml of 0.01 M phosphate-buffered saline (PBS, pH 7.4). This perfusion was followed by administration of 500 ml of 4% paraformaldehyde in 0.1 M phosphate buffer (PB, pH 7.4). The L5 spinal segment was removed and postfixed in the same fixative for 2 h at 4 °C. Fixed specimens were then cryoprotected in 30% sucrose in 0.1 M PB for 12 h at 4 °C. The spinal segments were cut into transverse sections (35 μm thick) with a freezing microtome (Kryostat 1720; Leitz, Mannheim, Germany) and were subsequently collected in dishes. Briefly, all of the sections were independently rinsed three times in 0.01 M PBS. Next they were incubated at room temperature (RT) for 30 min with 10% (v/v) fetal bovine serum (FBS; F6178; Sigma, St Louis, MO) in 0.01 M PBS containing 0.3% Triton X-100. Sections from the different dishes were subsequently incubated at room temperature overnight with one of the following antibodies: (1) goat-anti Iba-1 antiserum (1:1000 dilution, ab5076; Abcam, Cambridge, MA); (2) rabbit-anti Fos antiserum (1:1000 dilution, ab7963; Abcam); (3) rabbit-anti Fos antiserum (1:1000 dilution; Abcam) and goat-anti Iba-1 antiserum (1:1000 dilution; Abcam); (4) rabbit-anti Fos antiserum (1:1000 dilution; Abcam) and mouse-anti NeuN antiserum (1:3000 dilution, MAB377; Millipore, Billerica, MA); (5) goat-anti Iba-1 antiserum (1:1000 dilution; Abcam) and mouse-anti p-P38 antiserum (1:1000 dilution, AB45381; Abcam). The antibodies were diluted to their working concentrations in 0.01 M PBS containing 5% (v/v) FBS, 0.3% (v/v) Triton X-100, 0.05% (w/v) NaN_3_ and 0.25% (w/v) carrageenan (PBS-FBS, pH 7.4). The sections were washed three times in 0.01 M PBS and then incubated for 2 h at RT with the following respective secondary antibodies (in PBS containing 0.3% Triton X-100): (1) Alexa Fluor 488 conjugated guinea pig anti-goat IgG (1:400 dilution; Molecular Probes, Eugene, Oregon); (2) Alexa Fluor 488 conjugated donkey anti-rabbit IgG (1:400 dilution; Molecular Probes); (3) Alexa Fluor 594 conjugated donkey anti-rabbit IgG (1:400 dilution; Molecular Probes) and Alexa Fluor 488 conjugated guinea pig anti-goat IgG (1:400 dilution; Molecular Probes); (4) Alexa Fluor 488 conjugated donkey anti-rabbit IgG (1:400 dilution; Molecular Probes) and Alexa Fluor 594 conjugated guinea pig anti-mouse IgG (1:400 dilution; Molecular Probes); (5) Alexa Fluor 488 conjugated donkey anti-goat IgG (1:400 dilution; Molecular Probes) and Alexa Fluor 594 conjugated guinea pig anti-mouse IgG (1:400 dilution; Molecular Probes). Following three separate PBS washes, all of the sections were mounted onto gelatin-coated glass slides and cover-slipped with a mixture of 5% (v/v) glycerin and 2.5% (w/v) triethylenediamine in 0.1 M PBS. The sections were observed with a confocal laser-scanning microscope (CLSM) (Fluoview 1000, Olympus, Tokyo, Japan), using laser beams of 543 and 488 nm with appropriate emission filters for Alexa 594 (593–615 nm) and Alexa 488 (510–525 nm), respectively. Digital images were captured using FLUOVIEW software (Olympus, Tokyo, Japan). To measure the area of Iba-1 immunopositive somata, we used the Threshold Image function in Measure of MetaMorph 6.1 to set the low and high thresholds for the immunofluorescent intensity which was determined to be a signal. For Fos and NeuN, we randomly selected five sections of 35-μm thickness from each rat (n = 4 rats; total 20 sections) and then counted the number of immunopositive cells. The numbers of counted cells were corrected by use of Aber-crombie’s equation: number of cell = number of cells counted×T/(T + h), where T = thickness of the sections and h = the mean diameter of the nuclei of the large or small cells.

A mixture of normal rabbit, goat, or mouse sera was used to replace the first specific rabbit, goat and mouse primary antibodies to incubate the sections from the dishes. The following staining procedures used the staining methodology outlined in this section. Immunopositive staining was not observed for these samples.

### Western blot analysis

Animals were anesthetized by intraperitoneal injection of sodium pentobarbital (60 mg/kg) in 0.9% (w/v) saline. The animals were then sacrificed by decapitation. Protein (50 μg) was extracted from ipsilateral dorsal part of L5 spinal horn from each animal. Tissues were homogenized in ice-cold lysis buffer (50 mM Tris, pH 7.4, 150 mM NaCl, 5 mM EGTA, 0.5% NP-40, 10 mM NaF, and 1 mM PMSF) and rotated at 4 °C for 1 h before the supernatant was extracted. The supernatant was extracted by centrifugation at 12,000 g at 4 °C for 5 min. Equal amounts of protein extracts were denatured and subjected to SDS–polyacrylamide gelelectrophoresis. After separation, proteins were transferred to nitrocellulose membranes (Bio-Rad). The membranes were blocked with 5% nonfat milk in TBST (25 mM Tris, pH 7.4, 137 mM NaCl, 2.7 mM KCl, and 0.05% Tween 20) for 1 h at room temperature and incubated with goat-anti-Iba-1 (1:1,000; Abcam) or rabbit-anti-Fos (1:1000; Abcam) overnight at 4 °C. Mouse-anti-β-actin (1:1,000; Sigma) was used as a loading control. After washing three times with TBST, bound primary antibodies were detected with horseradish peroxidase-conjugated secondary antibodies (anti-goat 1:3,000, anti-mouse 1:5,000, anti-rabbit 1:3,000; Amersham Pharmacia Biotech Inc., Piscataway, NJ, USA). Between each step, the immunoblots were rinsed with Tris-buffered saline containing 0.02% Tween-20 (TBST). All reactions were detected by the enhanced chemiluminescence (ECL) detection method (Amersham). The densities of chemiluminescent bands from the protein blots were scanned and analyzed using Labworks Software (Ultra-Violet Products, UK) and normalized to β-actin levels.

### Enzyme-Linked Immunosorbent Assay (ELISA)

Concentrations of IL-1β, IL-6, and BDNF in the spinal cord were detected by ELISA at day 9. Rats were sacrificed and the lumbar segments of spinal cord were collected and stored at −80 °C until sonication. Total protein was dissociated mechanically from tissue using an ultrasonic cell disruptor, and then centrifuged at 3,000 × g for 15 minutes. Supernatant was removed and stored at −20 °C until analysis. IL-1β, IL-6, and BDNF protein were quantified using an enzyme-linked immunosorbent assay kit according to the manufacturer’s protocol (RapidBio Lab, Calabasas, CA). Measurement was completed using an enzyme-linked immunosorbent assay with an absorbency maximum at 450 nm.

### Statistical analysis

The immunofluorescent intensity of Iba-1, the number of Fos positive or NeuN positive cells and the protein levels of Iba-1 and Fos were analyzed using two-way analysis of variance (ANOVA) across groups. ELISA and Mechanical allodynia data were analyzed using two-way ANOVA (groups by days). Students-Newman-Keuls method was used as a post hoc test to detect differences between groups. All data were expressed as means ± S.E.M. All tests were two-sided and statistical significance was defined as P < 0.05.

## Results

### SPS + CFA-induced mechanical allodynia was attenuated following minocycline or c-*fos* ASO treatment

**Figure 2:**
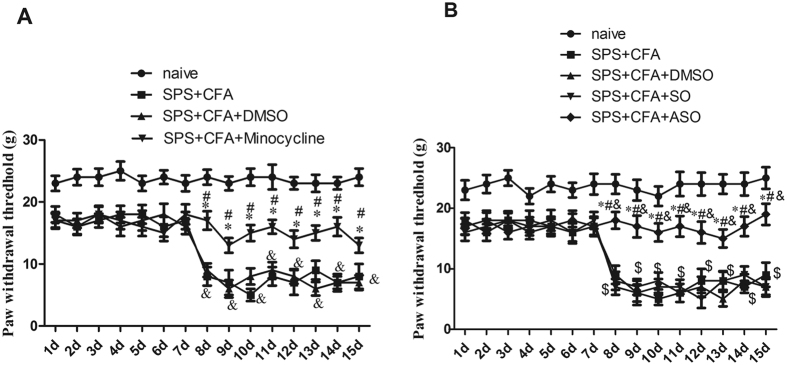
(**A**) Comparison of CFA + SPS and chronic Minocycline administration in relation to mechanical hyperalgesia. SPS + CFA-exposed rats had significantly induced mechanical hyperalgesia as shown by the Von Frey tests. The paw withdrawal threshold (PWT) was reduced in the injured hindpaw of the SPS + CFA-exposed rats compared with the naïve rats. Minocycline reversed the PWT reduction (*P < 0.05, compared to the SPS + CFA group; ^#^P < 0.05, compared to the SPS + CFA + saline group; ^&^P < 0.01, compared to the naïve group). (**B**) Comparison of CFA + SPS and chronic ASO administration in relation to mechanical hyperalgesia. SPS + CFA-exposed rats had significantly induced mechanical hyperalgesia as shown by the von Frey tests. The paw withdrawal threshold (PWT) was reduced in the injured hindpaw of the SPS + CFA-exposed rats compared to the naïve rats. ASO reversed the PWT reduction. There was no significant difference in the occurrence of mechanical hyperalgesia between the SO-treated groups and the DMSO-treated groups (*P < 0.05, compared to the SPA + CFA group; ^#^P < 0.05, compared to the SPS + CFA + DMSO group; ^$^P < 0.05, compared to the SPA + CFA + SO group; ^&^P < 0.01, compared to the naïve group).

Previous results showed that PWTs were significantly reduced in the SPS group from day seven (P < 0.05), the CFA group after CFA injection (P < 0.05), and the SPS + CFA-exposed group (P < 0.01) from day seven compared to the naïve rats. PWT was further reduced in injured hindpaws of the SPS + CFA-exposed rats from day 9 after CFA injection compared to CFA-exposed rats (P < 0.05)^7^. In the present study, there was a significant effect (A, F_2,105_ = 95.72, P < 0.001; B, F_3,140_ = 84.33, P < 0.001) on the paw withdrawal thresholds (PWTs). Chronic minocycline treatment markedly increased the PWT in SPS + CFA-exposed rats (P < 0.05) (Fig. 2A). Chronic minocycline treatment markedly increased the PWT in SPS-exposed rats or CFA-exposed rats (P < 0.05) (Supplemental Fig. 1A). Treatment with the neuron inactivator, c-*fos* ASO, reversed mechanical allodynia induced by SPS + CFA (P < 0.05) (Fig. 2B). Treatment with the ASO reversed mechanical allodynia induced by SPS or CFA (P < 0.05) (Supplemental Fig. 1B). Additionally, the effect of DMSO injection was not significantly different from the SO group (P > 0.05) (Fig. 2B).

### SPS + CFA-exposed rats showed significant upregulation of Iba-1 and Fos in the spinal cord dorn horn

**Figure 3:**
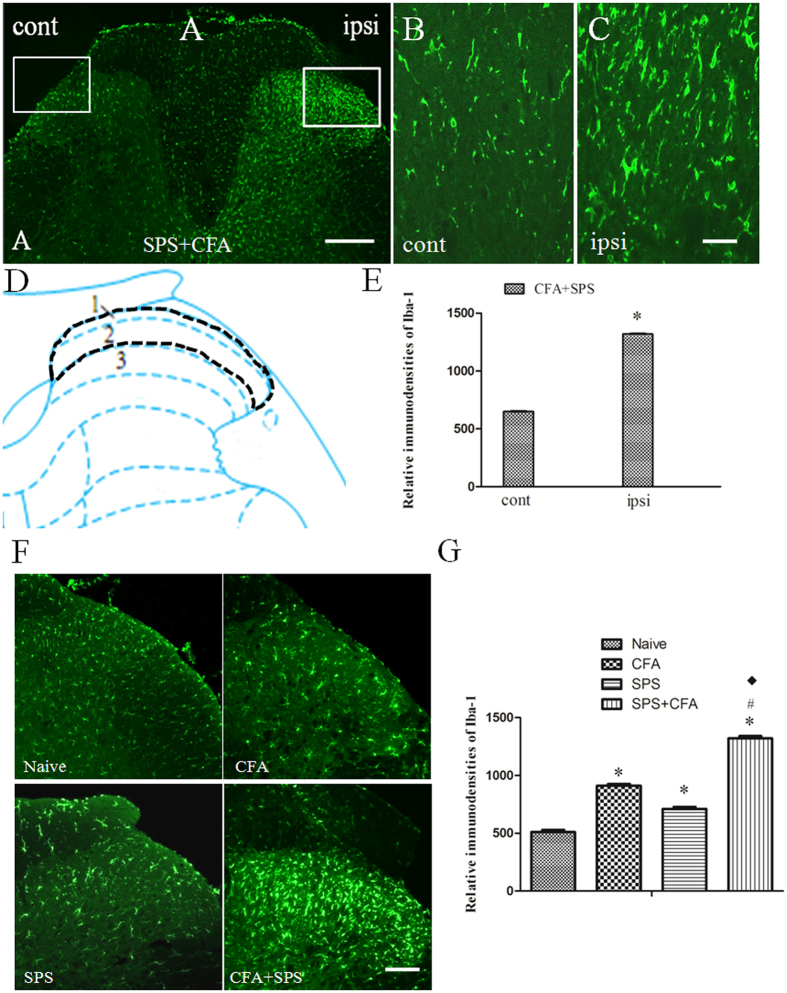
Immunofluorescent staining for Ionized calcium-binding adapter molecule-1 (Iba-1) in the spinal dorsal horn after SPS + CFA (**A**–**G**). (**B**,**C**) are magnified images of the rectangles represented in the panels in (**A**). (**D**) Scheme showing an overview of detected region (laminae 1, 2). E, SPS + CFA induced a significant upregulation in Iba-1 expression in the ipsilateral dorsal horn (*P < 0.05, compared to the contralateral dorsal horn). Scale bar, 200 μm (**A**) and 80 μm (**B**,**C**). (**F**) Representative microphotographs depicting the expression of Iba-1 on day 9 in each group. (**G**) Comparison of relative immunodensities of Iba-1 in different groups. Columns represent means ± S.E.M. of relative immunodensities of Iba-1 in the spinal dorsal horn (*P < 0.05, compared to the naïve group; ^#^P < 0.05, compared to the CFA group; ^◆^P < 0.05, compared to the SPS group). Scale bars, 100 μm (**G**).

**Figure 4:**
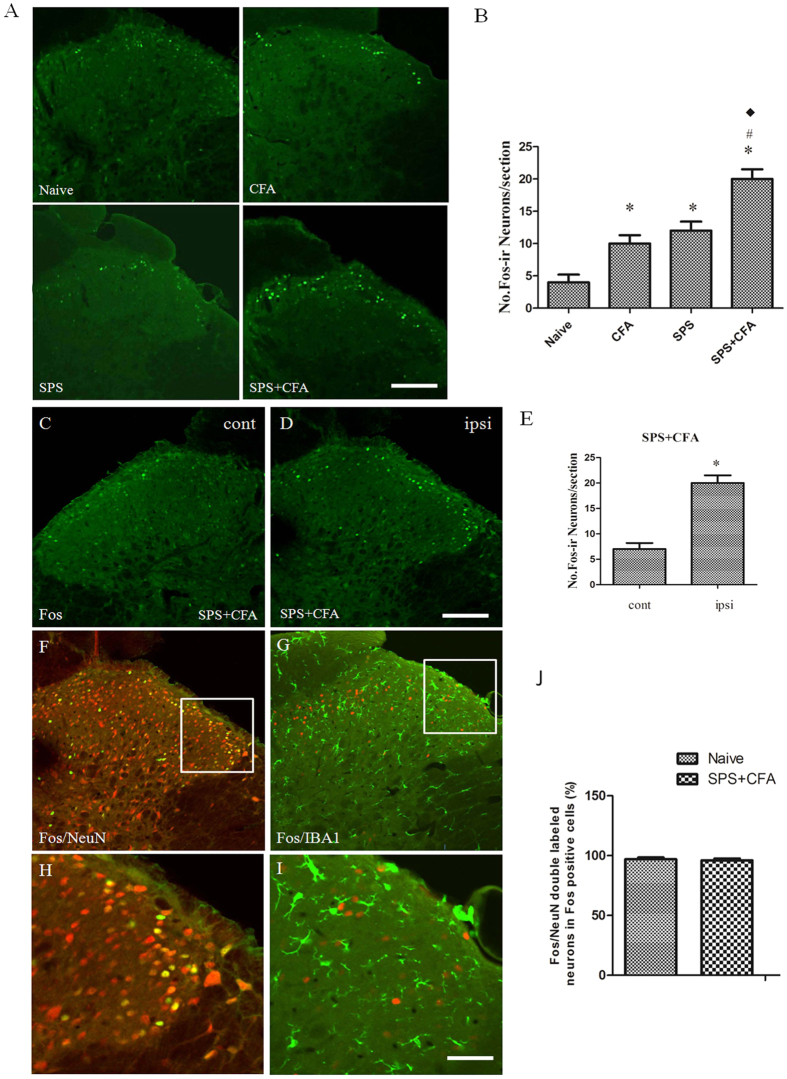
Double immunofluorescence analysis for Fos/NeuN and Fos/Iba-1 in the ipsilateral dorsal horn after SPS + CFA. Representative microphotographs depicting the expression of Fos on day 9 in each group (**A**). (**B**) Comparison of No. Fos-ir neurons/section in different groups. Columns represent means ± S.E.M. of No. Fos-ir neurons/section in the spinal dorsal horn (*P < 0.05, compared to the naïve group; ^#^P < 0.05, compared to the CFA group; ^◆^P < 0.05, compared to the SPS group). Scale bars, 100 μm (**A**). C-E SPS + CFA induced a significant upregulation of Fos expression in the ipsilateral dorsal horn (*P < 0.05, compared to the contralateral dorsal horn). Scale bar, 200 μm (**C**,**D**). (**H**,**I**) represent magnified images of the rectangles represented in the top panels in (**F**,**G**), respectively. J Statistical results showed the proportion of Fos/NeuN double-labeled neurons in total Fos-positive cells.

**Figure 5:**
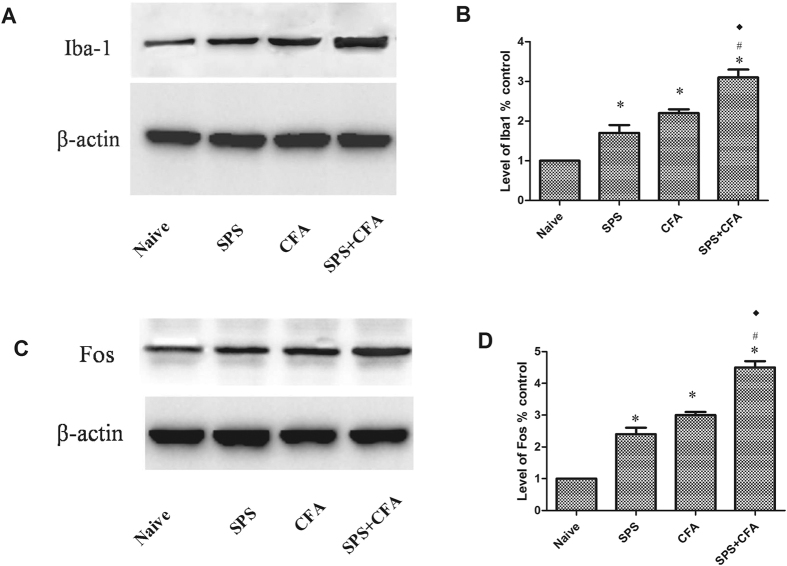
(**A**,**C**) Immunoblots of Iba-1 or Fos in the spinal dorsal horn in each group. Each well was loaded with 50 μg of total protein. (**B**,**D**) Densitometry analysis of bands corresponding to Iba-1 or Fos. Compared to the naïve group, Iba-1 was up-regulated at the protein level in SPS-exposed rats, CFA-exposed rats and SPS + CFA-exposed rats. Furthermore, Iba-1 was up-regulated in SPS + CFA-exposed rats compared to CFA-exposed rats or SPS-exposed rats (*P < 0.05, compared to the naïve; ^#^P < 0.05, compared to the CFA group; ^◆^P < 0.05 compared to the SPS group). Compared to the naïve group, Fos was up-regulated at the protein level in SPS-exposed rats, CFA-exposed rats and SPS + CFA-exposed rats. Furthermore, Fos was up-regulated in SPS + CFA-exposed rats compared to CFA-exposed rats or SPS-exposed rats (*P < 0.05, compared to the naïve; ^#^P < 0.05, compared to the CFA group; ^◆^P < 0.05 compared to the SPS group).

**Figure 6:**
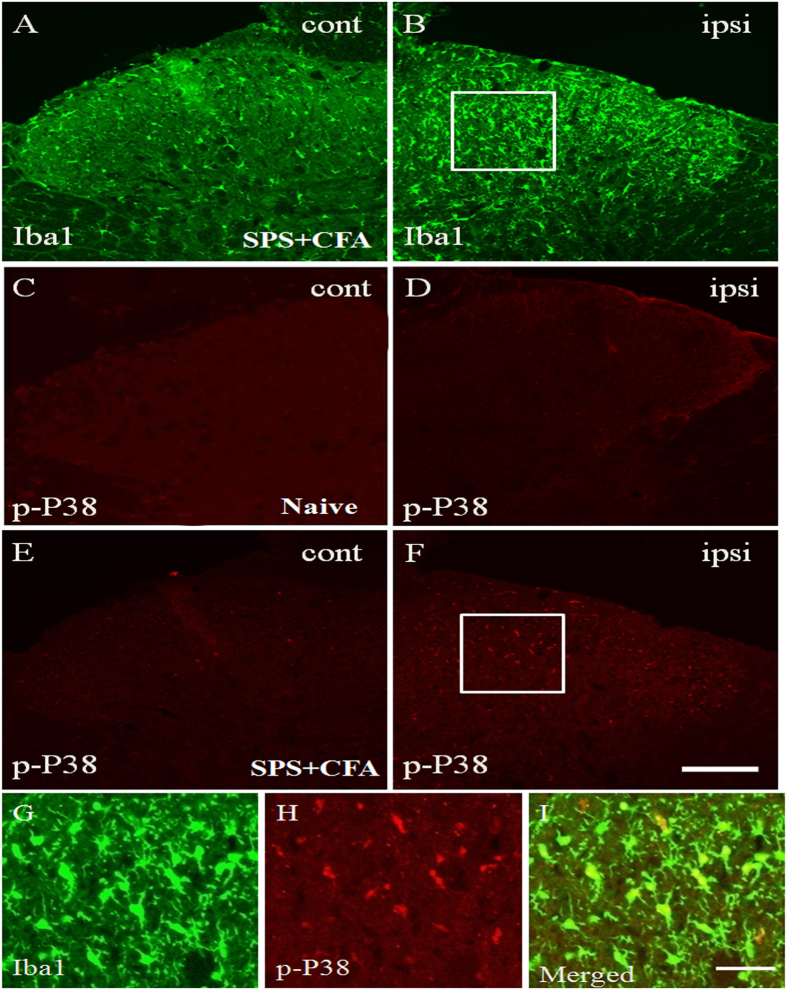
Microglial phosphorylated P38 (p-P38) activation contributes to SPS + CFA-induced mechanical allodynia. SPS + CFA increase expression of the Iba1 (**A**,**B**) and p-P38 (**E**,**F**) in the ipsilateral but not contralateral spinal dorsal horn. Double immunofluorescence analysis shows that p-P38 is completely co-localized with Iba1 (**G**–**I**). (**G**,**H**) are the magnified images of the rectangles represented in the panels in (**B**,**F**), respectively. Scale bars = 300 μm in (**A**–**F**) and 50 μm in (**G**–**I**).

In order to determine whether the CFA + SPS model presented with activated microglia and whether this response was exacerbated by stress, we monitored immunoreactivity for the microglial marker, Iba-1, in the spinal cord dorn horn. The CFA + SPS model induced morphological indices of microglial reactivity in the ipsilateral but not the contralateral dorsal horn (P < 0.05) (Fig. 3A–E). There were significant differences between groups in relation to the fluorescence intensity of Iba-1 (F_3,12_ = 69.75, P < 0.001). Morphological analysis showed that Iba-1 was up-regulated in both SPS-exposed or CFA-exposed and SPS + CFA-exposed rats compared with naïve rats (P < 0.05). In addition, increased levels of Iba-1 expression were observed in SPS + CFA-exposed rats compared with CFA- or SPS-exposed rats (P < 0.05) (Fig. 3F,G). Increased Fos expression levels were observed in SPS + CFA-exposed rats compared with CFA- or SPS-exposed rats (P < 0.05) (Fig. 4A,B). Additionally, CFA + SPS induced a significant increase in Fos expression in the ipsilateral but not the contralateral spinal dorsal horn (Fig. 4C–E). Almost all of the Fos-IR cells were positive for NeuN (97% of Fos-IR neurons were NeuN-immunoreactive positive, Fig. 4F). CFA + SPS-induced Fos expression was restricted to the neuronal population. This observation was made following calculation of the ratio of Fos positive NeuN-IR cells to Fos-IR cells. This ratio did not vary significantly (Fig. 4J). Thus, increased Fos expression was reliably used as a marker for activated neurons but not microglia. Double immunofluorescent labeling indicated that few Iba1-IR cells were positive for Fos, while most of them contained overlapping nuclei (which is symptomatic of activated neurons (Fig. 4F,H). The activated microglia and Fos protein were predominantly distributed in the superficial laminae of dorsal horn ipsilateral, suggesting a role in the nociceptive transmission. The Fos-IR neurons were surrounded by Iba1-IR processes, forming a large number of close contacts (Fig. 4G,I). The levels of Iba-1 and Fos in the spinal cord dorn horn were also analyzed (Fig. 5). There were significant differences in Iba-1 and Fos protein levels in the spinal cord dorn horn between groups (Fig. 5A, F_3, 12_ = 138.7, P < 0.001; Fig. 5B, F_3, 12_ = 30.83, P < 0.001). Western blot analysis showed that Iba-1 was upregulated in the SPS-, CFA- and SPS + CFA-exposed rats compared with naïve rats (P < 0.05) (Fig. 5A). In addition, Iba-1 levels were upregulated in the SPS + CFA-exposed rats compared with the CFA-exposed rats (P < 0.05) and SPS-exposed rats (P < 0.05) (Fig. 5B). Western blot analysis showed that Fos was upregulated in the SPS-,CFA- and SPS + CFA-exposed rats compared with naïve rats (P < 0.05) and Fos levels were upregulated in the SPS + CFA-exposed rats compared with CFA-exposed rats (P < 0.05) (Fig. 5C,D). The CFA + SPS model induced activation of P38 (indicated by phosphorylation) in the ipsilateral spinal dorsal horn (Fig. 6E,F). Following double immunofluorescence analysis, Phospho-P38 (p-P38) was found to be completely co-localized with Iba-1 in the spinal cord (Fig. 6G–I), but rarely co-localized with NeuN or GFAP (Supplemental Fig. 4).

### C-*fos* ASO or minocycline treatment down-regulated SPS + CFA-induced neuronal and microglial activation

**Figure 7:**
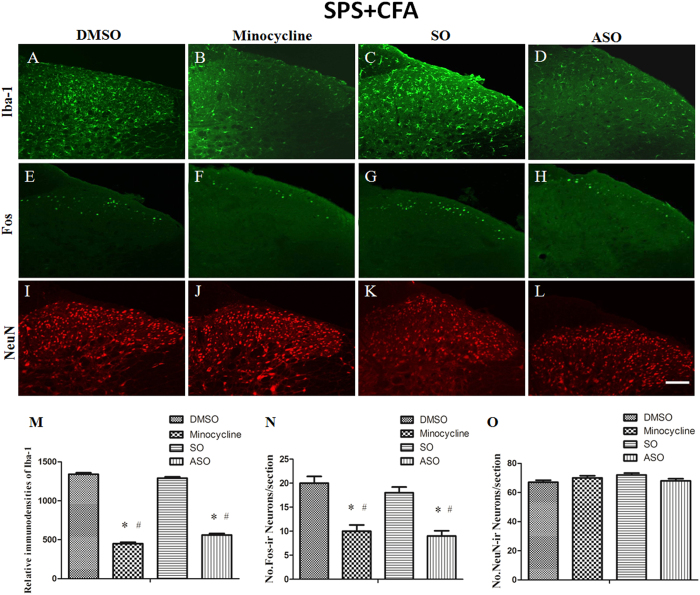
The expression of Fos and Iba-1 in the ipsilateral dorsal horn in DMSO, minocycline, c-*fos* SO and ASO groups after SPS + CFA. Compared to the DMSO-treated group, minocycline produced a significant inhibitory effect on microglial activation (**A**,**B**), as well as on Fos activation (**F**) in the spinal dorsal horn. Compared to DMSO- or the c-*fos* SO-treated group, c-*fos* ASO produced a significant inhibitory effect on Fos protein expression (**E**,**G**,**H**), as well as on microglial activation (**D**) in the spinal dorsal horn. (**M**,**N**) Statistical results showed of No. Fos-ir neurons/section or relative immunodensities of Iba-1 was compared between the different groups (*P < 0.05, compared to the DMSO group; ^#^P < 0.05, compared to the SO group). There were no significant differences between groups in relation to the number of NeuN-IR cells (**I**–**L**,**O**).

In order to determine whether the activated microglia and neurons are involved in “crosstalk” regulatory interactions that contribute to PTSD-induced hyperalgesia, we observed the effect of intrathecal (i.t.) injection of c*-fos* ASO or Minocycline on neuron and microglia activation morphologically. Our analysis revealed that treatment resulted in significant effects (Fig. 7A–D, F_3,20_ = 25.65, P < 0.001; Fig. 6E–H, F_3,20_ = 131.3, P < 0.001). Minocycline significantly inhibited CFA + SPS-induced Iba1 and Fos expression in the ipsilateral dorsal horn. Minocycline-treated rats showed a striking decrease in activated microglia compared to the DMSO-treated group (P < 0.05) (Fig. 7A–D). Minocycline-treated rats showed a striking decrease in activated neurons compared to the DMSO-treated group (P < 0.05) and the SO-treated group (P < 0.05) (Fig. 7E–H). c-*fos* ASO significantly inhibited CFA + SPS-induced Fos and Iba1 expression in the ipsilateral dorsal horn. c-*fos* ASO-exposed rats showed a striking decrease in activated microglia compared to the DMSO-treated group (P < 0.05) and the SO-treated group (P < 0.05) (Fig. 7A–D). Meanwhile, c-*fos* ASO-treated rats showed a striking decrease in activated neurons compared to the DMSO-treated group (P < 0.05) and the SO-treated group (P < 0.05) (Fig. 7E–H). In addition, there was no significant difference in the numbers of NeuN-IR cells between the groups (Fig. 7I–L).

### IL-6 mediates crosstalk between activated microglia and neurons

**Figure 8:**
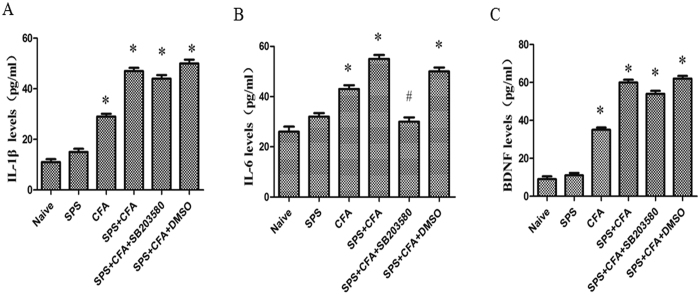
Comparison of CFA + SPS and chronic SB203580 administration on IL-1 (**A**), IL-6 (**B**) and BDNF (**C**) production by ELISA. Compared to the naïve group, CFA + SPS induced an increase in IL-1β, IL-6, and BDNF production. SB203580 administration reversed the increase in IL-6 production (*P < 0.05, compared to the naïve group; ^#^P < 0.05, compared to the SPS + CFA group).

**Figure 9:**
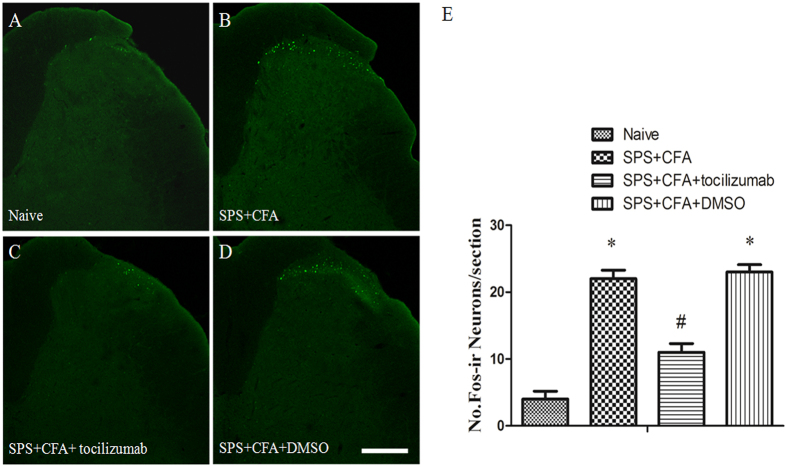
Comparison of CFA + SPS and chronic tocilizumab administration on the expression of Fos. Compared to the CFA + SPS-treated group, tocilizumab produced a significant inhibitory effect on Fos activation (*P < 0.05, compared to the naïve group; ^#^P < 0.05, compared to the SPS + CFA group).

In order to determine whether cytokine involved in crosstalk between activated microglia and neurons, we observed CFA + SPS on IL-1β, IL-6, and BDNF production. CFA + SPS induced a striking increase in IL-1β, IL-6, and BDNF production compared to naïve group (P < 0.05) (Fig. 8). However, intrathecal administration of SB203580, a specific P38 inhibitor, only reversed the CFA + SPS-induced increase in IL-6 production, but not IL-1β and BDNF. Moreover, intrathecal administration of tocilizumab, an IL-6 receptor (IL-6R) inhibitor, could show a striking decrease in activated neurons compared to SPS + CFA-treated rats (Fig. 9). Our double immunofluorescent study further demonstrated that in addition to NeuN-positive neurons IL-6R (gp130) could also co-localize with part of GFAP or Iba-1 positive cells in the back horn of the spinal cord (Supplemental Fig. 5).

## Discussion

Previous studies conducted by our research group have demonstrated that exposure to PTSD can enhance CFA-induced allodynia^7^. Both CFA- and SPS + CFA-treated animals demonstrated mechanical hyperalgesia compared to the control group. SPS + CFA-treated animals also presented with significantly higher levels of mechanical hyperalgesia compared to CFA-treatment animals. The present study produced the following findings: (1) SPS + CFA-treated animals displayed mechanical hyperalgesia compared to the control group. This result is consistent with our previous studies. (2) We observed increased immunoreactivity towards Iba-1 in spinal cord sections and elevated spinal levels of Iba-1 following immunofluorescence and Western Blot analysis on samples attained from stressed rats and CFA-treatment animals. In addition, a further increase in Iba-1 activation was observed in the spinal dorsal horn after SPS + CFA treatment compared with SPS or CFA treatment. (3) We observed elevated spinal levels of Fos following immunofluorescence and Western Blot analysis on samples attained from CFA-treatment animals. In addition, a further increase in Fos activation in the spinal dorsal horn was observed after SPS + CFA treatment compared with CFA treatment. Almost all of the Fos-IR cells stained positive for NeuN. (4) We observed that p-P38 was co-localized with Iba-1 in the spinal cord following immunofluorescence. (5) Both C-*fos* ASO and Minocycline treatment resulted in the down-regulation of SPS + CFA-induced neuronal and microglial activation. In addition, chronic administration of either Minocycline or C-*fos* ASO attenuated the combined model-induced hyperalgesia. (6) Inhibition of P38 activation by SB203580 suppressed IL-6 production and inhibition IL-6 receptor (IL-6R) activation by tocilizumab suppressed Fos activation.

Several reports have demonstrated that stress results in the activation of inflammatory processes^14^,^25^,^26^. Acute stress, such as restraint or swimming, leads to antinociception^27^. However, chronic stressful stimuli have been shown to induce an increase in pain sensitivity^7^. PTSD is a chronic stress and anxiety disorder, which can increase pain sensitivity^28^. However, the precise cellular and molecular mechanism that underpins PTSD-induced hyperalgesia in the spinal dorsal horn is poorly understood. Evidence indicates that neuroinflammation has been implicated in the induction of chronic pain. Therefore, we speculated that microglia would exist in a hyperactivated state in stressed rats^29^. To test this hypothesis, we used CFA, SPS, and SPS + CFA combined animals in this study to understand the mechanisms that trigger this condition. Emerging evidence suggests that stress may affect immune responses within the CNS. Recent research has also suggested that stress can potentiate inflammatory responses to promote peripheral immune stimulation under certain conditions^12^. In this study, we found that SPS results in microglial activation in the spinal dorsal horn. These data are in agreement with studies demonstrating that stress affects immune responses. This might suggest that microglial activation is one of the factors that facilitate SPS-induced hyperalgesia.

Previous studies have shown that chronic restraint stress in mice induces brain microglial activation and proliferation. Additionally, restraint stress in rats has been shown to induce microglial activation in the hippocampus^30^. Our results showed that microglial activation may be related to PTSD and to the spinal site. SPS could lead to the secretion of hormones, e.g., glucocorticoid hormone^28^. Glucocorticoid receptors (GRs) are expressed on microglia in culture. Considerable evidence suggests that microglial cells are targets for glucocorticoids (GCs), and it has been suggested that the former respond differently depending on the duration and the context of exposure^3^,^31^. Our results show that SPS + CFA-treated animals produce significantly higher levels of Iba-1 activation than CFA-treated or SPS-treated animals in the spinal dorsal horn. An increase in Iba-1 activation may account for the occurrence of stress-induced hyperalgesia.

It is likely that activated microglia contribute to an enhanced pain-like state through microglia-neuron communication. Our studies show that there is a more pronounced increase in Fos activation in the spinal dorsal horn after SPS + CFA treatment than CFA treatment. Additionally, many Fos-IR neurons were observed to be surrounded by Iba1-IR processes that had formed many close contacts. This form of contact suggests a possible morphological basis for a regulatory “crosstalk” between activated microglia and neurons. Intrathecal administration of minocycline or C-*fos* ASO attenuated SPS + CFA-induced mechanical allodynia. Morphological results showed that C-*fos* ASO or Minocycline treatment down-regulated SPS + CFA-induced neuronal and microglial activation. These results further showed that SPS induced dysregulation of immune responses and Iba-1 activation. It is possible that these effects might lead to CFA-induced pain sensitivity. It is likely that activated microglia contribute to the enhanced pain-like state through microglia-neuron communication. It is feasible that GCs facilitate microglia-neuron communication.

Our previous results demonstrated that activation of extracellular signal-regulated kinase1/2 (ERK1/2) in the medial prefrontal cortex may contribute to stress-induced hyperalgesia^7^. Other studies suggested that stress-enhanced allodynia was associated with increased dorsal horn extracellular signal-regulated kinase phosphorylation (pERK)^14^. In the present study, we showed that activated P38 co-localized with Iba-1, not co-localized with GFAP or NeuN. It suggested that stress accelerated the microglial response to CFA dependent on the activation of P38 signaling. Inhibition of P38 activation by SB203580 suppressed IL-6 production, not BDNF and IL-1 production. Microglial activation through activation of P38 leads to increased synthesis of IL-6. Our morphological results showed that almost all of the IL-6-IR was positive for Iba1. Direct modulation of dorsal horn neuronactivity by IL-6 may be involved in the development of PTSD-induced hyperalgesia. Previous studies showed that IL-6 receptor gp130 was expressed in rat sensory neurons and immune and myeloid cells^32^. Gp130 is associated protein of IL-6R, so we use gp130 as the marker of IL-6 signaling pathway. Our results showed that IL-6R (gp130) co-localized with part of NeuN, Iba-1 and GFAP positive-cells after SPS + CFA treatment. Moreover, inhibition IL-6 receptor (IL-6R) activation by tocilizumab suppressed Fos activation in SPS + CFA. It is feasible that this activation promoted the release of IL-6, facilitating microglia-neuron interaction in PTSD-induced hyperalgesia. However, we cannot rule out the possibility that IL-6 may also serve as an autocrine or paracrine factor to activate microglia themselves or activated astrocytes to facilitate hyperalgesia in our case. So, further study is needed to address the precise function and signaling mechanisms of IL-6 in SPS + CFA-induced hyperalgesia.

Together, our findings demonstrate that PTSD-induced dysregulation of immune responses could lead to an increase in mechanical allodynia. Hyperalgesia induced in our SPS + CFA rats model is accompanied by elevated Iba-1 and Fos activation. IL-6 might be essential for the crosstalk between activated microglia and neurons in the modulation of SPS + CFA-induced hyperalgesia.

## Additional Information

**How to cite this article**: Qi, J. *et al*. Crosstalk between Activated Microglia and Neurons in the Spinal Dorsal Horn Contributes to Stress-induced Hyperalgesia. *Sci. Rep.* **6**, 39442; doi: 10.1038/srep39442 (2016).

**Publisher's note:** Springer Nature remains neutral with regard to jurisdictional claims in published maps and institutional affiliations.

## Supplementary Material

Supplementary Information
