# Supplementary material for: Crosstalk between Activated Microglia and Neurons in the Spinal Dorsal Horn Contributes to Stress-induced Hyperalgesia
Source: Sci Rep. 2016 Dec 20;6:39442. doi: 10.1038/srep39442 (PMC5171842; doi:10.1038/srep39442)
Supplement: Supplementary Information [file srep39442-s1.pdf]

Crosstalk between Activated Microglia and Neurons in the Spinal Dorsal Horn Contributes to Stress-induced Hyperalgesia

Jian Qi[1#], Chen Chen[3], Qing-Xi Meng[1], Yan Wu[2], Haitao Wu[2, 4#], Ting-Bao Zhao[1#]

[1]Department of Spinal Cord Injury and Rehabilitation, The General Hospital of Jinan Military Command, Jinan 250031, China

[2]Department of Neurobiology, Institute of Basic Medical Sciences, Beijing 100850, China

[3]Department of Pharmacy, The Second Hospital of Shandong University, Jinan, 250031, China

[4] Co-innovation Center of Neuroregeneration, Nantong University, Nantong, Jiangsu, 226001, China.

Supplemental methods

The Morris water maze (MWM)

The Morris water maze (MWM) is one of the most widely used tasks in behavioral neuroscience for studying learning. The MWM apparatus is made up of a black colored pool (160 cm in diameter and 55 cm in height) and divided into four quadrants. The pool was filled with 20-23℃ water. A cylindrical dark platform was fixed in one of the quadrants (the target quadrant). The platform was submerged about 2 cm below the surface of the water during the spatial learning trials. Four extra-maze cues were placed on the wall in the vicinity of the pool. Swim activity in four quadrants was recorded by a digital video camera positioned directly above the pool. At first, animals were put into a quadrant (not containing the platform). If rats could not find platform within 60 s, help was provided to get the rats onto the platform and keep them there for 20 s. Rats were trained four times each day, with a 20-s interval between trials. The escape latency (calculated by averaging four trial values) to find the platform was used as a measure of learning performance. Rats were tested for seven consecutive days for learning performance.

The elevated plus-maze (EPM)

The elevated plus-maze (EPM) test consists of a plus-shaped platform located 45 cm above the floor. There are closed arms and open arms. At the beginning of the test, rats were placed in the central area (10×10 cm) of the maze, facing an enclosed arm (Shanghai Mobiledatum Information Technology Co., Ltd, Shanghai China). The rats' activity was recorded by a video camera in 5 min. Anxiety in the plus-maze is indicated by an increase in the percentages of time spent in the open arms (time in open arms/total time in all arms), and an increase in the percentages of entries into the open arms (entries into open arms/total entries into all arms).

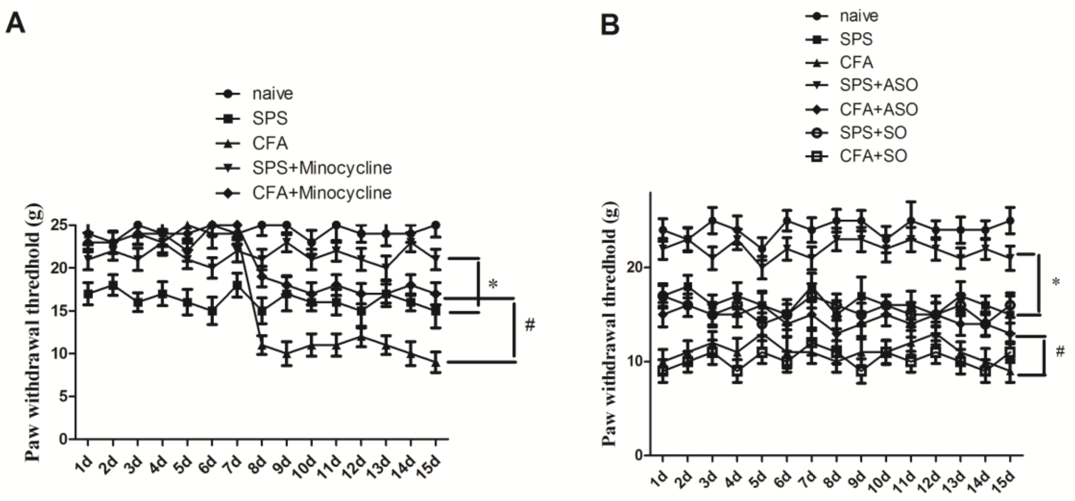

Supplemental Figure 1 A Comparison of CFA, SPS and chronic Minocycline administration in relation to mechanical hyperalgesia. SPS or CFA-exposed rats had induced mechanical hyperalgesia as shown by the Von Frey tests. The paw withdrawal threshold (PWT) was reduced in the injured hindpaw of the SPS or CFA-exposed rats compared with the naïve rats. Minocycline reversed the PWT reduction (* P<0.05, compared to the SPS group; # P<0.05, compared to the CFA group). **B** Comparison of CFA or SPS and chronic ASO administration in relation to mechanical hyperalgesia. SPS- or CFA-exposed rats had induced mechanical hyperalgesia as shown by the von Frey tests. The paw withdrawal threshold (PWT) was reduced in the injured hindpaw of the SPS- or CFA-exposed rats compared to the naïve rats. ASO reversed the PWT reduction (* P<0.05, compared to the SPS group; # P<0.05, compared to the CFA group).

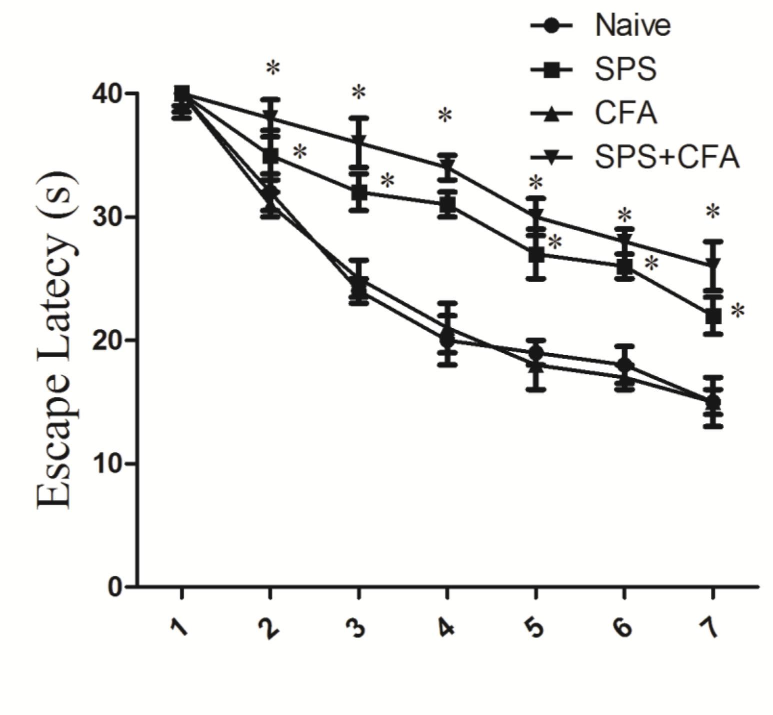

Supplemental Figure 2 Comparison of CFA, SPS, SPS+CFA on mean escape latency. Line graph showing the differences of the mean escape latency among groups in the MWM test (* P<0.05, compared to naïve rats).

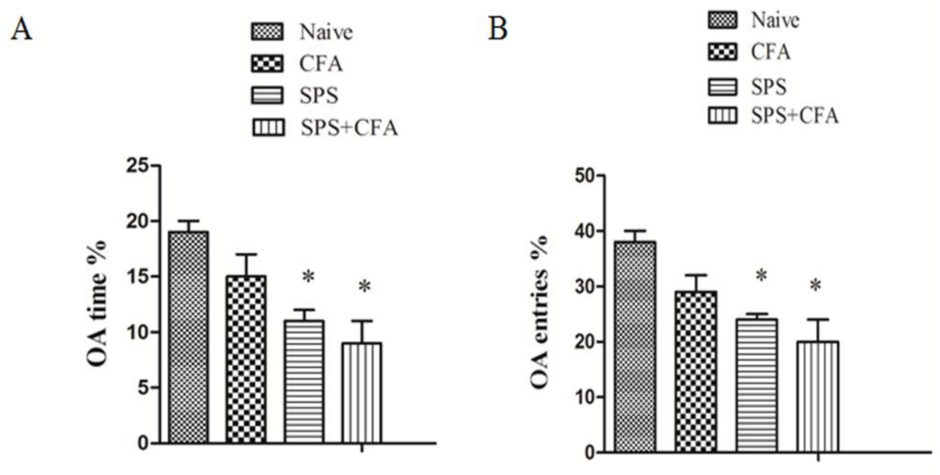

Supplemental Figure 3 The percentage of open-arm time (open arm (OA) time/total time). Histogram showing the comparison of open-arm (OA) time/total time among groups in the EPM test (*P<0.05, compared to naïve rats).

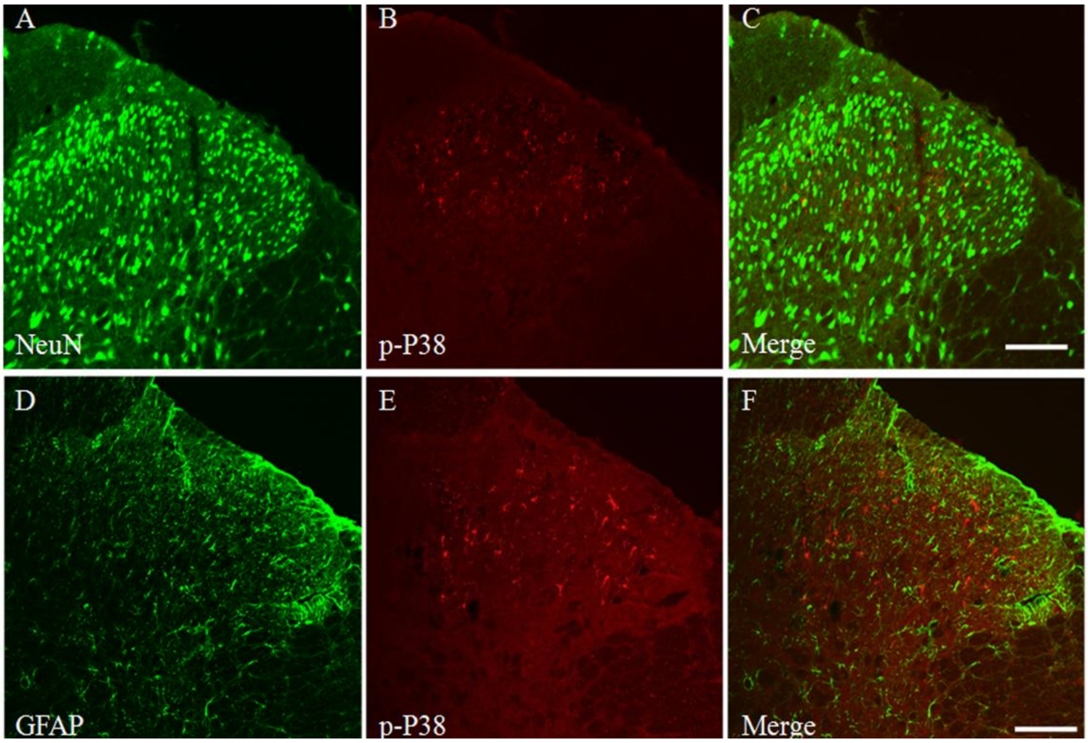

Supplemental Figure 4 Double immunofluorescence analysis showed that p-P38 was rarely co-localized with NeuN (A–C) or GFAP (D-E) after SPS+CFA. Scale bars = 250 μm in A–F.

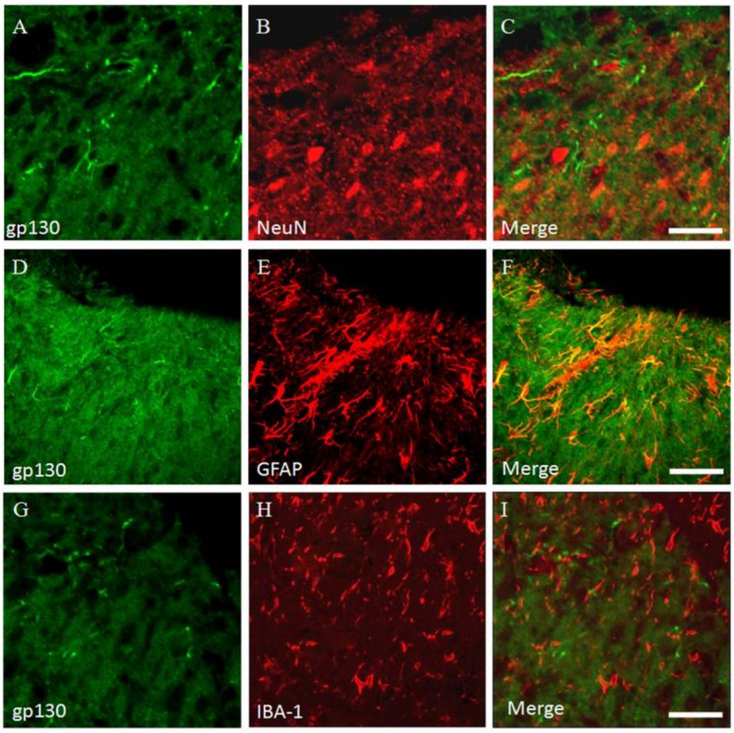

Supplemental Figure 5 Double immunofluorescence analysis for gp130/NeuN (A-C), gp130/GFAP (D-F), and gp130/Iba1(G-I) in the ipsilateral dorsal horn after SPS+CFA. Scale bars = 30 μm in A–I.
